# Association between Ocular Bacterial Carriage and Follicular Trachoma Following Mass Azithromycin Distribution in The Gambia

**DOI:** 10.1371/journal.pntd.0002347

**Published:** 2013-07-25

**Authors:** Sarah E. Burr, John D. Hart, Tansy Edwards, Ignatius Baldeh, Ebrima Bojang, Emma M. Harding-Esch, Martin J. Holland, Thomas M. Lietman, Sheila K. West, David C. W. Mabey, Ansumana Sillah, Robin L. Bailey

**Affiliations:** 1 Department of Clinical Research, Faculty of Infectious and Tropical Disease, London School of Hygiene and Tropical Medicine, London, United Kingdom; 2 Medical Research Council Unit, Fajara, Banjul, The Gambia; 3 MRC Tropical Epidemiology Group, London School of Hygiene and Tropical Medicine, London, United Kingdom; 4 Francis I. Proctor Foundation for Research in Ophthalmology, University of California San Francisco, San Francisco, California, United States of America; 5 Dana Center for Preventive Ophthalmology, Johns Hopkins University, Baltimore, Maryland, United States of America; 6 National Eye Health Programe, Ministry of Health and Social Welfare, Kanifing, The Gambia; University of Cambridge, United Kingdom

## Abstract

**Background:**

Trachoma, caused by ocular *Chlamydia trachomatis* infection, is the leading infectious cause of blindess, but its prevalence is now falling in many countries. As the prevalence falls, an increasing proportion of individuals with clinical signs of follicular trachoma (TF) is not infected with *C. trachomatis*. A recent study in Tanzania suggested that other bacteria may play a role in the persistence of these clinical signs.

**Methodology/Principal Findings:**

We examined associations between clinical signs of TF and ocular colonization with four pathogens commonly found in the nasopharnyx, three years after the initiation of mass azithromycin distribution. Children aged 0 to 5 years were randomly selected from 16 Gambian communitites. Both eyes of each child were examined and graded for trachoma according to the World Health Organization (WHO) simplified system. Two swabs were taken from the right eye: one swab was processed for polymerase chain reaction (PCR) using the Amplicor test for detection of *C. trachomatis* DNA and the second swab was processed by routine bacteriology to assay for the presence of viable *Streptococcus pneumoniae*, *Haemophilus influenzae*, *Staphylococcus aureus* and *Moraxella catarrhalis*. Prevalence of TF was 6.2% (96/1538) while prevalence of ocular *C. trachomatis* infection was 1.0% (16/1538). After adjustment, increased odds of TF were observed in the presence of *C. trachomatis* (OR = 10.4, 95%CI 1.32–81.2, p = 0.03), *S. pneumoniae* (OR = 2.14, 95%CI 1.03–4.44, p = 0.04) and *H. influenzae* (OR = 4.72, 95% CI 1.53–14.5, p = 0.01).

**Conclusions/Significance:**

Clinical signs of TF can persist in communities even when ocular *C. trachomatis* infection has been controlled through mass azithromycin distribution. In these settings, TF may be associated with ocular colonization with bacteria commonly carried in the nasopharnyx. This may affect the interpretation of impact surveys and the determinations of thresholds for discontinuing mass drug administration.

## Author Summary

Trachoma, the world's leading infectious cause of blindness, is caused by ocular infection with the bacterium *Chlamydia trachomatis*. In low-prevalence settings and following mass treatment campaigns, clinically active follicular trachoma (TF) can be found in the absence of *C. trachomatis* infection. We carried out this study to investigate associations between ocular carriage of non-chlamydial pathogens and a clinical diagnosis of TF following a mass treatment campaign in The Gambia. We found that children who carried *Streptococcus pneumoniae* or *Haemophilus influenza* in their eyes were more likely to have been diagnosed with TF than children who did not carry these pathogens. In The Gambia, non-chlamydial pathogens may be inducing or exacerbating TF in the absence of *C. trachomatis* infection.

## Introduction

Trachoma, caused by ocular infection with the intracellular bacterium *Chlamydia trachomatis*, remains the leading infectious cause of blindness world-wide. In 1986, The Gambia's first national blindness survey indicated that trachoma was a significant cause of blindness in the country with active trachoma affecting 17% of children aged 0–14 years and very high rates of trichiasis in those 15 years and older [1], [2]. In response to these findings, the National Eye Health Programme (formerly the National Eye Care Programme), supported by SightSavers International, established a network of Community Ophthalmic Nurses (CONs) trained and equipped to screen communities for active trachoma and to conduct trichiasis surgery. Since the 1980s, many trachoma endemic communities may have also benefited from water and sanitation interventions such as well digging and latrine construction programmes via the Departments of Water Resources and Community Development. A Healthy Eyes curriculum, focused on preventive eye care, was introduced into primary schools and a network of individuals (*Nyateros* or ‘Friends of the Eye’) trained in eye health and ocular first aid was established at the community level.

A decade later, the 1996 national survey found a substantially reduced prevalence of active trachoma (to just over 5% in 0–14 year olds nationally), but which remained above 10% in four regions of the country [2], [3]. In 2006, in the absence of a national survey, trachoma surveys were conducted in two regions (North Bank and Lower River Regions) and showed little change in overall trachomatous inflammation, follicular (TF) prevalence from 1996 [4]. On the basis of these data, The Gambia's plan for trachoma control using mass drug administration (MDA) with azithromycin was approved by the Director of Medical Services in 2006 and The Gambia received over 400,000 doses of azithromycin from the International Trachoma Initiative (ITI) to be distributed in 23 priority health districts. The Partnership for the Rapid Elimination of Trachoma trial (PRET) [5], [6] was embedded in this plan, with communities in four priority districts randomized to receive treatment annually for three years [as currently recommended by the World Health Organization (WHO)] or to stop treatment if the prevalence of TF or detected ocular *C. trachomatis* infection fell below 5%. The PRET study areas now have very low rates of both disease and *C. trachomatis* infection [7].

As the prevalence of TF has fallen in The Gambia, a disparity between clinical signs of disease and ocular *C. trachomatis* infection has been documented [4], [6], [8]. This finding has been mirrored in other communities following implementation of mass treatment [9]–[12] and in communitites where prevalence of TF is low [13]–[16]. It has been suggested that at least some of the TF now observed may be due to organisms other than *C. trachomatis* [4], [16]. In the present study, we aimed to investigate possible associations between ocular colonization with four bacterial pathogens commonly found in the nasopharnyx and a clinical diagnosis of TF following the implementation of the MDA campaign in The Gambia.

## Methods

### Ethics statement

Ethical approval was obtained from the London School of Hygiene and Tropical Medicine Ethics Committee and The Gambia Government/Medical Research Council Unit, The Gambia Joint Ethics Committee under study numbers SCC 1107v2 and L2011.25. Oral consent was obtained from the community leaders and informed written (thumbprint or signature) consent was obtained from each child's guardian at the time of examination. The use of thumbprints in the consent procedure was specifically approved by the ethics committees. The consent form was further signed by an independent witness.

### Study design

The PRET trial (ClinicalTrials.gov NCT00792922) was a cluster randomized trial (CRT) carried out in The Gambia, the design of which has been described elsewhere [5]. Briefly, clusters, or enumerations areas (EAs, population 600–800 individuals), were randomly sampled from four districts (two districts in the North Bank Region, two districts from the Western Region; 12 EAs per district). Twenty-four EAs received three annual, community-wide azithromycin treatments and 24 EAs received just one mass treatment at baseline. The 48 EAs were further randomized in a 2×2 factorial design to receive standard treatment coverage (one day visit to EAs by treatment team) versus enhanced coverage (two visits to each EA) during the mass treatments that took place. Randomization of EAs was stratified by district (six EAs per district allocated to either three mass treatments or one). The study described here is ancillary to the PRET trial; 16 of the 24 EAs from North Bank Region were included in this study (8 from each frequency allocation arm, Fig.? 1).

**Figure 1:**
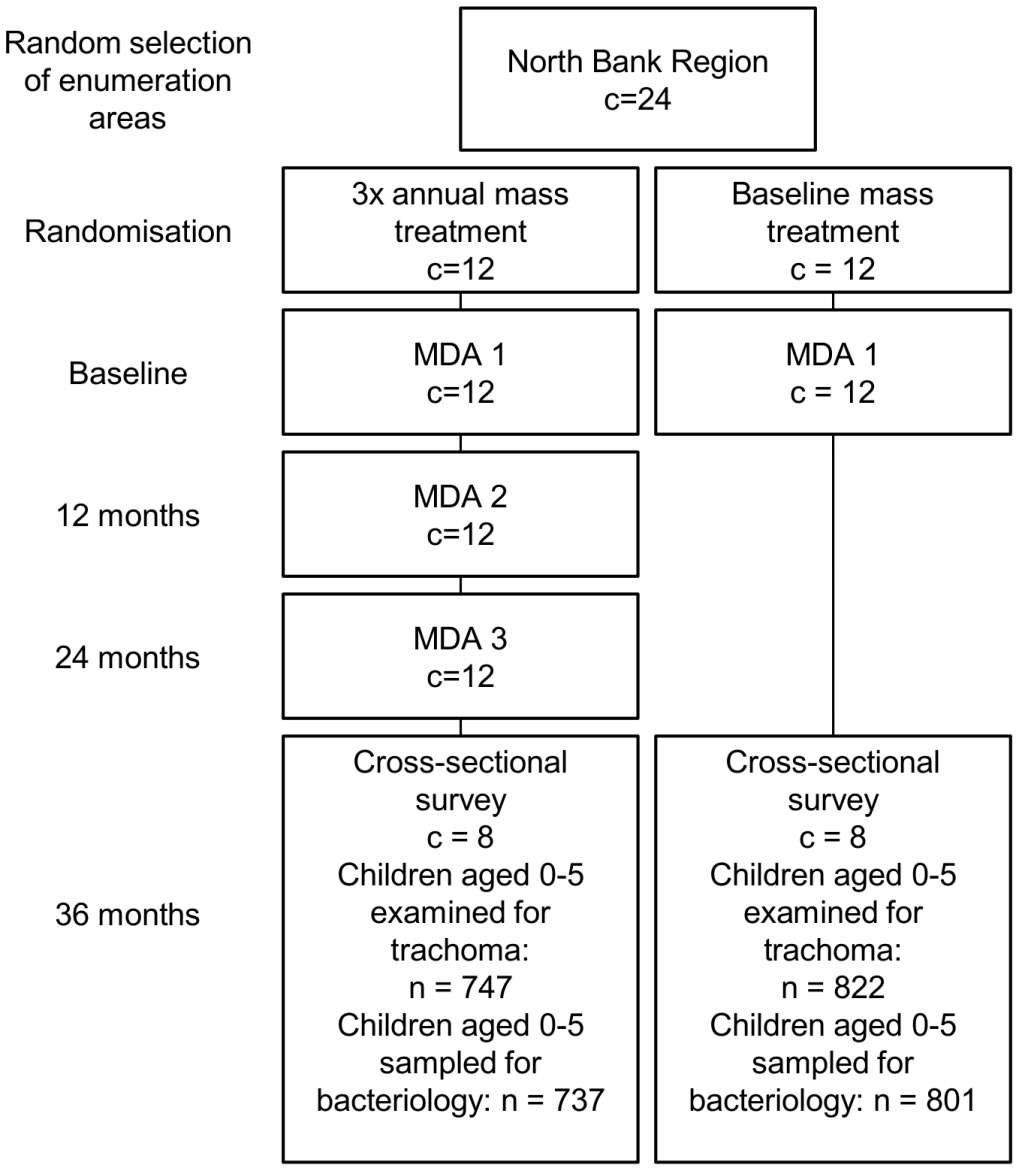
Study allocation.

### Field methods

A cross-sectional survey took place during May and June 2011, coinciding with the 36 month follow-up survey of the PRET trial. For EAs randomized to receive three annual rounds of mass treatment, sample collection took place 12 months following the last round; for EAs randomized to receive a single treatment round, sample collection took place 36 months following treatment (Fig.? 1). One hundred children aged 0–5 years were randomly selected from each EA for participation. Eligible children were: resident in the EA, did not have an ocular condition precluding examination or specimen collection, willing to have a sample taken and had a guardian willing to provide consent. Children's faces were initially observed for the presence of nasal and ocular discharge and flies on the eyes. The upper eyelids of each child were everted and the tarsal conjunctiva graded for signs of clinical trachoma using the WHO simplified grading system [17]. Two photographs were then taken of the right everted upper tarsal conjunctiva for use in quality assurance as detailed elsewhere [7]. Samples were collected from the upper tarsal conjunctiva and lower fornix of the right eye using a Dacron swab. Upper tarsal conjunctival swabs were placed into dry vials while lower fornix swabs were placed into vials containing 1 ml skimmed-milk-tryptone-glucose glycerol transport medium (STGG) [18]. Samples were kept on wet ice in the field and frozen at −20°C within 10 hours. Samples were transferred to −70°C upon arrival at the MRC laboratory, within 2 weeks' time.

### Laboratory methods

Upper tarsal conjunctival samples were processed for *C. trachomatis* infection using the Amplicor CT/NG kit (Roche Molecular Systems, Branchburg, NJ, USA) as previously described [5]. Lower fornix swabs were thawed at room temperature and cultured on blood and chocolate agar plates for the detection of *Streptococcus pneumoniae*, *Haemophilus influenzae*, *Staphylococcus aureus* and *Moraxella catarrhalis*. Blood agar plates were incubated at 37°C in either 5% CO_2_ or under anaerobic conditions. Chocolate agar plates were incubated at 37°C in 5% CO_2_. Isolates were identified using conventional bacteriology techniques. All laboratory personnel were masked to the clinical trachoma grade.

### Sample size

A sample size of 1568 subjects is required to detect an odds ratio (OR) of 2 for a univariate association between TF (anticipated to be 7%) and a bacterial pathogen (assumed to be present in 15% of participants without TF), assuming 80% power to detect univariate associations with 95% confidence. It was expected that a sample size of approximately 1600 children would be achieved from 16 EAs in which a random sample of 100 children was to be taken from each.

### Analysis methods

Overall unadjusted prevalence is presented with corresponding exact binomial 95% confidence intervals (CI). Prevalence is also presented adjusted for age, sex and district using random effects logistic regression models to account for any underlying variation detected between households and between EAs.

The prevalence of TF was tabulated by each pathogen, age, sex, district, *C. trachomatis* infection and time since mass treatment (12 months for those in the annual treatment arm versus 36 months for those in the baseline treatment arm). Clustering of TF and bacterial pathogens at household and EA level was investigated using random effects logistic regression models with random intercepts for household and EAs. Likelihood ratio tests (LRTs) were used to compare models with and without the additional random effects terms.

Univariate associations with TF were determined using random effects logistic regression models with and without the exposure of interest. To assess confounding of the association between each pathogen and TF, unadjusted Mantel-Haenszel ORs for the effect of each pathogen on TF were compared to the ORs adjusted for each exposure. A 10% change in the OR was considered to indicate confounding, if the exposure was associated with both the pathogen of interest and TF. Modification of the effect of each pathogen on TF by other exposures was assessed in exploratory analyses using Mantel-Haenszel methods.

Following identification of univariate associations with TF and confounders of the effect of each pathogen on TF, a multivariate model was built adjusting for each pathogen associated with TF and including any confounding exposures. Independent risk factors for TF were also considered for inclusion in the final model on the basis of a LRT p-value<0.1. The strength of association with TF for each pathogen and exposure in the final model was estimated from a LRT comparing adjusted models with and without the pathogen of exposure.

Predictors of discharge (nasal and ocular) were identified using random effects logistic regression with discharge as the outcome, accounting for between household and/or between EA variation where appropriate and building a final multivariable model using the same approach as for TF.

## Results

In the 16 EAs under study, 1569 children aged 0–5 years were randomly sampled for measurement of trachoma outcomes as part of the PRET CRT. Of these, laboratory results for both *C. trachomatis* PCR and conventional bacteriology were obtained for 1538 children (98.0%). For the 31 children with missing laboratory results, tabulations showed no evidence that children were not missing at random. Analysis was restricted to the results obtained for those 1538 children with complete data.

### Prevalence of bacterial colonization

Bacteriology culture detected at least one non-chlamydial pathogen in the eyes of 475 (30.9%) children. *S. aureus* and *S. pneumoniae* were most commonly detected occuring in more than 14% of children (Table? 1). Colonization with *H. influenzae* and *M. catarrhalis* was less common. Sixteen children (1.0%) had evidence of *C. trachomatis* infection.

**Table 1:** Prevalence of ocular bacterial colonization, *C. trachomatis* infection and TF.

	N	Colonization, n	Prevalence (exact binomial 95% CI, percent scale)	Adjusted Prevalence* (95% CI, percent scale)
*S. pneumoniae*	1538	219	14.2 (12.5–16.1)	12.5 (11.0–13.8)
*H. influenzae*	1538	57	3.7 (2.8–4.8)	2.6 (1.6–3.6)
*S. aureus*	1538	233	15.2 (13.4–17.0)	14.7 (13.9–15.6)
*M. catarrhalis*	1538	19	1.2 (0.7–1.9)	1.1 (0.8–1.5)
*C. trachomatis*	1538	16	1.0 (0.6–1.7)	1.0 (0.8–1.1)
TF	1538	96	6.2 (5.1–7.6)	6.1 (5.4–6.9)

* Adjusted for age (0–1, 2–3, 4–5 years), sex, district and any underlying variation between households and/or EAs as appropriate.

Dual colonization was detected in 53 (3.4%) children; most commonly with *S. pneumoniae* and *S. aureus* (37 children, 2.4%). Dual colonization with *S. pneumonaie* and *H. influenza* was found in 10 children (0.7%). Six children positive for *C. trachomatis* infection carried one non-chlamydial pathogen concurrently (0.4%); 2 children had *C. trachomatis* and *H. influenzae*, 2 children had *C. trachomatis* with *S. aureus* and 2 children had *C. trachomatis* and *M. catarrhalis*. No children were found to have *C. trachomatis* infection concurrent with *S. pneumoniae*.

In random effects logistic regression adjusting for each pathogen alone (null models), there was evidence that children carrying *S. pneumoniae* were clustered at the household level as demonstrated by evidence of between-household variation (p<0.001) but not at EA level, after accounting for clustering by household (p = 0.11). *H. influenzae* carriage also clustered at household level (p = 0.01) but not at EA level (p = 0.46). The reverse was found for *M. catarrhalis* carriage, which clustered at EA level (p = 0.01) but not household (p = 1.00). *S. aureus* carriage clustered at both household and EA levels (p<0.001). *C. trachomatis* infection cases were clustered at EA level (p<0.001).

### Associations with TF

Ninety-six children (6.2%) were found to have clinical signs of TF; no children were diagnosed as having trachomatous inflammation – intense (TI). TF cases were clustered at both EA and household levels (p<0.001). After accounting for between-household and between-EA variation, age (p = 0.004), *C. trachomatis* infection (p = 0.04) and district (p = 0.08) were associated with TF in univariate analyses (Table? 2). Sex (p = 0.64), time since mass treatment was carried out in the EA (whether 12 or 36 months previously; p = 0.76) and flies on the eyes (p = 0.317) were not associated with TF.

**Table 2:** Associations between bacterial pathogens and TF in 0–5 year olds.

Characteristic		N	TF, n (%)	Univariate OR (95% CI)*	p-value	Multivariate OR (95% CI)*	p-value
All children		1538	96 (6.2)	-	-	-	
*S. pneumoniae*	No	1319	78 (5.9)	1		1	
	Yes	219	18 (8.2)	1.69 (0.85–3.33)	0.140	2.14 (1.03–4.44)	0.044
*H. influenzae*	No	1481	88 (5.9)	1		1	
	Yes	57	8 (14.0)	3.40 (1.20–9.66)	0.027	4.72 (1.53–14.5)	0.009
*S. aureus*	No	1305	81 (6.2)	1		-	
	Yes	233	15 (6.4)	1.30 (0.63–2.67)	0.480	-	
*M. catarrhalis*	No	1519	96 (6.3)	-		-	
	Yes	19	0 (0)	-	-	-	
At least one non-chlamydial pathogen	No	1063	60 (5.6%)	1		-	
	Yes	475	36 (7.6%)	1.93 (1.09–3.43)	0.022	-	
Number of non-chlamydial pathogens	0	1036	60 (5.6%)	1	0.072	-	
	1	422	31 (7.4%)	1.96 (1.07–3.57)		-	
	2	53	5 (9.4%)	1.79 (0.51–6.31)		-	
Age (years)	0–1	464	17 (3.7)	1	0.012	1	0.004
	2–3	588	47 (8.0)	2.72 (1.36–5.43)		3.24 (1.55–6.75)	
	4–5	486	32 (6.6)	1.89 (0.93–3.87)		2.46 (1.14–5.32)	
Sex	Male	820	54 (6.6)	1		-	
	Female	718	42 (5.9)	0.89 (0.53–1.47)	0.637	-	
*C. trachomatis*	No	1522	93 (6.1)	1		1	
	Yes	16	3 (18.8)	8.54 (1.21–60.4)	0.041	10.4 (1.32–81.2)	0.033
District	Lower Baddibu	726	30 (4.1)	1		1	
	Central Baddibu	812	66 (8.1)	2.25 (0.95–5.34)	0.075	2.32 (0.94–5.70)	0.076
Time since mass treatment	12 months	737	44 (6.0)	1		-	
	36 months	801	52 (6.5)	1.16 (0.45–2.99)	0.757	-	
Flies on the eyes†	No	227	16 (7.1)	1		-	
	Yes	1309	80 (6.1)	1.44 (0.71–2.90)	0.317	-	

* Univariate and multivariate ORs are estimated from random effects logistic regression models accounting for between-household and between-EA variation.

† 2 missing values for flies on the eyes.

In the univariate analysis accounting for between-household and between-EA variation of TF but no other covariates, increased odds of TF were observed in the presence of *S. pneumoniae* (OR = 1.69, 95%CI 0.85–3.33, p = 0.14), *H. influenzae* (OR = 3.40, 95%CI 1.20–9.66, p = 0.027) and *S. aureus* (OR = 1.30, 95%CI 0.63–2.67, p = 0.48) but a significant effect was observed only for *H. influenzae* in this analysis (Table? 2). There were no signs of TF amongst any of the children with ocular *M. catarrhalis* carriage. Dual carriage with more than one non-chlamydial pathogen was not associated with TF (p = 0.072).

The presence of *S. pneumoniae* was associated with age (p<0.001), with fewer older children having the organism. A similar association was found between *H. influenzae* and age (p<0.001). After adjustment for age, the association between *S. pneumoniae* and TF was significant at the 5% level (OR = 2.08, 95%CI 1.01–4.30, p = 0.05). The effect of *H. influenzae* also appeared stronger after adjustment for age (OR = 4.45, 95%CI 1.44–13.7, p = 0.01). *C. trachomatis* infection was not associated with age (p = 0.54).

In building a final random effects regression model accounting for between-household and between-EA variation, *H. influenzae* and *S. pneumoniae* were added in turn, followed by age since this was identified as a confounder of the effect of both *H. influenzae* and *S. pneumoniae* on TF. *C. trachomatis* infection and district were also added to the model in turn as independent risk factors for TF.

Multivariable results suggest a two-fold increase in the odds of TF for children with ocular *S. pneumoniae* carriage and a four-fold increase for carriers of *H. influenzae* (Table? 2). *C. trachomatis* infection was significantly associated with TF, but estimates of the magnitude of effect were imprecise due to low prevalence of *C. trachomatis* infection and TF in the sample.

### Predictors of nasal and ocular discharge

Overall, 839 (54.6%) children were observed to have nasal discharge on the day of screening. In the final regression model, after adjustment for underlying variation at household level, *S. pneumoniae* (p = 0.02), *M. catarrhalis* (p = 0.01) and TF (p = 0.05) were associated with increased odds of nasal discharge (Table? 3). Age was also associated with nasal discharge (p<0.001) with increased odds observed for 2–3 year olds versus 0–1 year olds and decreased odds for 4–5 year olds versus 0–1 year olds. In the univariate analysis, flies on the eyes were associated with nasal discharge (p<0.001) but we have not included this variable in the multivariate model as it is not clear whether nasal discharge is the casual effect for flies on the eyes.

**Table 3:** Associations with nasal discharge in 0–5 year olds.

Characteristic		N	Nasal discharge, n (%)	Univariable OR (95% CI)*	p-value	Multivariable OR (95% CI)*	p-value
All children		1538	839 (54.6)	-	-	-	
*S. pneumoniae*	No	1319	700 (53.1)	1		1	
	Yes	219	139 (63.5)	1.59 (1.15–2.20)	0.005	1.52 (1.08–2.13)	0.015
*H. influenzae*	No	1481	808 (54.6)	1		-	
	Yes	57	31 (54.9)	0.99 (0.55–1.78)	0.965	-	
*S. aureus*	No	1305	715 (54.8)	1		-	
	Yes	233	124 (53.2)	0.94 (0.69–1.28)	0.698	-	
*M. catarrhalis*	No	1519	824 (54.3)	1		1	
	Yes	19	15 (79.0)	3.58 (1.09–11.8)	0.022	4.07 (1.22–13.6)	0.013
TF	No	1442	776 (53.8)	1		1	
	Yes	96	63 (65.6)	1.67 (1.03–2.72)	0.034	1.63 (0.99–2.68)	0.051
*C. trachomatis*	No	1522	827 (54.3)	1		-	
	Yes	16	12 (75.0)	2.67 (0.75–9.49)	0.114	-	
Age (years)	0–1	464	260 (56.1)	1	<0.001	1	<0.001
	2–3	588	368 (62.6)	1.36 (1.03–1.79)		1.40 (1.06–1.85)	
	4–5	486	211 (43.4)	0.57 (0.43–0.75)		0.59 (0.44–0.79)	
Sex	Male	820	455 (55.5)	1		-	
	Female	718	384 (53.5)	0.90 (0.72–1.21)	0.342	-	
District	Lower Baddibu	726	393 (54.1)	1		-	
	Central Baddibu	812	446 (54.9)	1.00 (0.79–1.28)	0.972	-	
Time since mass treatment	12 months	737	395 (53.6)	1		-	
	36 months	801	444 (55.4)	1.10 (0.86–1.41)	0.433	-	
Flies on the eyes†	No	227	65 (28.6)	1		-	
	Yes	1309	634 (48.4)	2.54 (1.78–3.60)	<0.001	-	

* Univariable and multivariable ORs are estimated from random effects logistic regression models accounting for between-household and between-EA variation, as appropriate.

† 2 missing values for flies on the eyes.

Ocular discharge was observed for 190 (12.4%) children. A final model adjusted for household and EA level clustering of cases. Results suggest increased odds of ocular discharge in children carrying *H. influenzae* (p = 0.06) and possibly in male children (p = 0.07) (Table? 4). Age was again associated with ocular discharge (p = 0.04) with the same pattern as for nasal discharge; increased odds in 2–3 year olds versus 0–1 year olds and decreased odds for 4–5 years versus 0–1 year olds.

**Table 4:** Associations with ocular discharge in 0–5 year olds.

Characteristic		N	Ocular discharge, n (%)	Univariable OR (95% CI)*	p-value	Multivariable OR (95% CI)*	p-value
All children		1538	190 (12.4)	-	-	-	-
*S. pneumoniae*	No	1319	166 (12.6)	1		-	
	Yes	219	24 (11.0)	0.85 (0.52–1.39)	0.502	-	
*H. influenzae*	No	1481	177 (12.0)	1		1	
	Yes	57	13 (22.8)	2.24 (1.09–4.60)	0.035	2.07 (1.00–4.31)	0.059
*S. aureus*	No	1305	163 (12.5)	1		-	
	Yes	233	27 (11.6)	0.97 (0.60–1.56)	0.884	-	
*M. catarrhalis*	No	1519	188 (12.4)	1		-	
	Yes	19	2 (10.5)	0.81 (0.17–4.00)	0.795	-	
TF	No	1442	172 (11.9)	1		-	
	Yes	96	18 (18.8)	1.49 (0.81–2.76)	0.213	-	
*C. trachomatis*	No	1522	187 (12.3)	1		-	
	Yes	16	3 (18.8)	1.86 (0.43–8.13)	0.426	-	
Age (years)	0–1	464	64 (13.8)	1	0.030	1	0.038
	2–3	588	82 (14.0)	1.06 (0.72–1.56)		1.11 (0.75–1.63)	
	4–5	486	44 (9.1)	0.63 (0.40–0.97)		0.66 (0.42–1.02)	
Sex	Male	820	113 (13.8)	1		1	
	Female	718	77 (10.7)	0.74 (0.53–1.04)	0.082	0.73 (0.52–1.03)	0.069
District	Lower Baddibu	726	85 (11.7)	1		-	
	Central Baddibu	812	105 (12.9)	1.18 (0.62–2.25)	0.615	-	
Time since mass treatment	12 months	737	77 (10.5)	1		-	
	36 months	801	113 (14.1)	1.40 (0.76–2.57)	0.288	-	
Flies on the eyes†	No	227	193 (85.0)	1		-	
	Yes	1309	1155 (88.2)	1.25 (0.80–1.96)	0.338	-	

* Univariable and multivariable ORs are estimated from random effects logistic regression models accounting for between-household and between-EA variation, as appropriate.

† 2 missing values for flies on the eyes.

## Discussion

The PRET trial documented a near disapperance of ocular *C. trachomatis* infection from a catchment area of greater than 65,000 people in the two years following the intitiation of MDA; biannual surveys carried out in the four study districts showed no evidence of ocular *C. trachomatis* infection 12 and 18 months post-MDA initiation and just 0.1% prevalence at 6 and 24 months [7]. Yet prevalence of TF over the same time period remained relatively high, reaching more than 10% in some EAs in the North Bank Region of the country. While one cannot rule-out episodes of *C. trachomatis* infection in the intervening months, it is difficult to see how *C. trachomatis* infection alone could sustain the clinical signs of disease during that time. Low-level exposure to *C. trachomatis* may not be detected by the Amplicor PCR method but it is unclear whether such low bacterial loads result in onward transmission of the microorganism.

In the 16 EAs included in this study, prevalence of ocular *C. trachomatis* infection 36 months post-MDA was 1% while prevalence of clinical signs of TF was 6%. While the association between active ocular *C. trachomatis* infection and TF was strong, it does not account for all the TF recorded. Clinical signs of TF were also associated with ocular *S. pneumoniae* and *H. influenzae* carriage. Both *S. pneumoniae* and *H. influenzae* are well known causes of bacterial conjunctivitis although not generally assocaited with follicular conjunctivitis [19]. It may be that localised inflammation caused by these microorganims recalls a follicular response in children who have previously been exposed to *C. trachomatis*. Alternatively, ocular carriage could predispose the eye to infection by *C. trachomatis* thereby inducing subsequent TF. In the present study, ocular carriage with *S. pneumoniae* was associated with nasal discharge while *H. influenzae* was associated with ocular discharge. *Musca sorbens*, which in some environments can act as the vector of trachoma, is more often found on the faces of children with ocular and nasal discharge than on children with no discharge [20], [21]; in our study flies on the eyes was associated with nasal discharge. If ocular colonization with these pathogens itself causes discharge, this may in turn increase the risk of *C. trachomatis* transmission.

One limitation of this study was that samples were kept frozen at −20°C for approximately two weeks before transport to the laboratory and subsequent storage at −70°C. Published data suggest these handling conditions are sufficient for cultivation of these respiratory pathogens [22], [23] however, we cannot discount the possibilty that storage conditions negatively impacted the viability of our samples resulting in an underestimate in ocular carriage prevalence. In addition, it is unclear whether the isolation of non-chlamydial, bacterial pathogens from the conjunctiva in a cross-sectional survey such as this is indicative of established ocular infection. A positive bacteriology culture may simply indicate transient carriage in the conjunctiva following introduction of bacteria by fingers or fomites. Indeed, nasopharyngeal (NP) carriage rates of *S. pneumoniae* in The Gambia are high with up to 90% of children under the age of five years being carriers [24], [25]. It is likely that children continually self-inoculate their eyes by touching their hands to their faces, possibly causing clinical signs of trachoma in children who have previously been exposed to *C. trachomatis*.

Our study has also shown that prevalence of ocular carriage of *S. aureus* is high, yet no association of this pathogen with TF was found and its carriage was not associated with either nasal or ocular discharge. Unlike the other pathogens detected, *S. aureus* is a part of the normal skin flora and imprecise sampling of the lower fornix could have introduced skin contamination into the swabs. However, the prevalence of ocular *S. aureus* carriage that we have detected (15%) is consistent with an earlier study carried out in Gambian communities where 11% of adults presenting for trichiasis surgery were found to carry the pathogen [15]. Another study detected *S. aureus* in the eyes of 30% of adults with trachomatous scarring and in 30% of healthy controls' however, the sample size of this study was small (13 cases and 7 controls [26]).

Previous studies on the role of non-chlamydial pathogens in trachoma have been conducted in The Gambia but with a focus on trachomatous scarring (TS) and trichiasis (TT) rather than on TF [15], [26]. Bacterial pathogens were more frequently isolated from eyes with TS than from control eyes however, this difference was not statistically significant [26]. In contrast, TT was significantly assocaited with bacterial pathogens in the eye, an association strengthened with increasing disease severity as measured by the number of lashes touching the eye [15], [26]. *S. pneumoniae* was the most common pathogen isolated from TT cases [26].

The role of non-chlamydial pathogens in TF has recently been studied in a trachoma endemic community in Tanzania, in this case prior to rather than post-MDA [16]. Prevalence of TF was higher than that reported here, with 13.7% of children under 10 years of age displaying signs of active disease. Prevalence of ocular *C. trachomatis* infection was also higher (5.3%) yet no association was seen between TF and a positive Amplicor PCR result. Ocular *S. pneumoniae* and *H. influenzae* carriage however, were both found to be associated with TF, consistent with our present findings.

The prevalence of ocular carriage of *S. pneumoniae* in the Tanzanian study was modestly lower than that which we report here (10.2% versus 14.2%). This may reflect the broader age distribution of children sampled in the Tanzanian study (all children under 10 years of age) as NP carriage rates are known to be higher in younger children. However, prevalence of NP carriage also appears to be lower in Tanzania than in The Gambia, although data are limited [27], [28]. The heptavalent pneumococal conjungate vaccine (PCV-7) was introduced into the Expanded Programme of Immunization (EPI) in The Gambia in August 2009; those children who were less than two years of age when recruited into our study were likely vaccinated. Available evidence however, suggests prevalence of NP carriage of *S. pneumonaie* in The Gambia is not affected by PCVs due to replacement with non-vaccine serotypes [25], [29]. Indeed we found younger children were more likely to carry *S. pneumoniae* than older children, consistent with data of NP carriage from this country indicating prevalence of carriage is assocaited with decreasing age [24].

*H. influenzae* carriage rates were markedly different between the two studies with *H. influenzae* type B (Hib) being isolated from 14% and non-Hib *H. influenzae* from 9% of children in the Tanzaian study. We found ocular *H. influenzae* colonization in just 3.7% of children sampled. Hib conjugate vaccine has been used in the Gambian EPI since 1997 and coverage rates are believed to be high [30]. Prevalence of NP Hib carriage in Western Gambia fell from 12% pre-vaccination to 0.25% five years following introduction of the vaccine into the EPI [31]. We therefore did not type for Hib in the present study. It is likely that high Hib vaccine coverage has contributed to the low levels of ocular *H. influenzae* carriage reported here; while Hib vaccination does not cross protect against other *H. influenzae* serotypes, there are only limited data to support the assertion that Hib vaccination results in serotype replacement by other capsulated strains in carriage [32]. Although we did not use molecular techniques to characterise the *H. influenzae* isolates, we speculate the majority of them would be unencapsulated (non-typeable) strains, which are unaffected by the Hib vaccine. Such strains are often associated with conjunctivitis [33] and recently were shown to account for most of the *H. influenzae* carried in the nasopharnyx of a cohort of Gambian infants [34]. Despite the lower prevalence of ocular *H. influenzae* carriage seen in The Gambia as compared to Tanzania, we also found an association between TF and this pathogen.

The Gambia is one of many countries incorporating MDA into its national trachoma control strategy. The ITI has distributed more than 250 million azithromycin treatments in 19 trachoma endemic countries and plans to be active in 42 countries by 2015. While MDA with azithromycin is associated with a significant decrease in pharyngeal carriage of *S. pneumoniae*, this effect is short-lived with carriage rates returning to baseline within two months following a single round of treatment [35], [36]. Repeated MDA at six or 12 month intervals also fails to affect prevalence of NP carriage of *S. pneumoniae* in the long-term [37]–[39]. The effect of MDA with azithromycin on carriage of *H. influenzae* is less well characterized, yet available data from The Gambia indicate pharyngeal carriage of the bacterium is unaffected by three doses (20 mg/kg) given over the course of two weeks [40]. It is therefore likely that as ocular *C. trachomatis* infection is controlled through mass azithromycin distribution, the contribution of these non-chlamydial pathogens to clinical signs of TF will become more apparent.

According to WHO policy, the prevalence of TF is currently the principal determinant for discontinuing MDA [41]. If non-chlamydial pathogens are inducing or exacerbating TF in the absence of *C. trachomatis* infection, communities may end up receiving unnecessary treatment. Furthermore, proposed criteria for trachoma elimination also relate to prevalence of clinical signs [42], [43] and as countries strive to reach their control targets, the information obtained from surveillance may be misleading. The role of these pathogens in the persistence of TF post-MDA therefore warrants further study; the key question is whether TF in these circumstances is likely to progress to conjunctival scarring and blindness.
